# Central pancreatectomy without anastomosis

**DOI:** 10.1186/1477-7819-7-67

**Published:** 2009-08-31

**Authors:** Michael Wayne, Siyamek Neragi-Miandoab, Franklin Kasmin, William Brown, Anil Pahuja, Avram M Cooperman

**Affiliations:** 1 The Pancreas and Biliary Center at Saint Vincent's Hospital, Manhattan, 170 West 12thStreet, Cronin 454, New York, NY 10011, USA

## Abstract

**Background:**

Central pancreatectomy has a unique application for lesions in the neck of the pancreas. It preserves the distal pancreas and its endocrine functions. It also preserves the spleen.

**Methods:**

This is a retrospective review of 10 patients who underwent central pancreatectomy without pancreatico-enteric anastomosis between October 2005 and May 2009. The surgical indications, operative outcomes, and pathologic findings were analyzed.

**Results:**

All 10 lesions were in the neck of the pancreas and included: 2 branch intraductal papillary mucinous neoplasms (IPMNs), a mucinous cyst, a lymphoid cyst, 5 neuroendocrine tumors, and a clear cell adenoma.

**Conclusion:**

Central pancreatectomy without pancreatico-enteric anastomosis for lesions in the neck and proximal pancreas is a safe and effective procedure. Morbidity is low because there is no anastomosis. Long term endocrine and exocrine function has been maintained.

## Introduction

In 1957, Guillemin and Bessot [1] described central pancreatectomy (CP) in a patient with chronic pancreatitis. Central pancreatectomy (CP) has since been used in select cases for treating pancreatitis, most often for benign and low grade malignant lesions in the neck of the pancreas [2-4]. Potential advantages of central pancreatectomy include preservation of endocrine, exocrine, and splenic function [3,5-7].

Benign or low-grade malignant lesions in the neck of the pancreas have been treated surgically, either by pancreaticoduodenectomy resection (PDR) or distal pancreatectomy with splenectomy (DPS) or splenic preserving distal pancreatectomy (SPDP). Each operation involves a resection of a major portion of the pancreas, which in a diseased pancreas can worsen diabetes mellitus and/or exocrine insufficiency [8,9]. This paper will discuss the technique and benefits of a resection of the central portion of the pancreas; a simplification of the procedure, and a literature review of the topic.

## Materials and methods

A review of patients who underwent CP between October 2005 and May 2009 at St. Vincent's Medical Center was done after approval by the Institutional Review Board. The mean age of patients was 54 ± 15 years and ranged from 34 to 77 years old. There were 5 male and 5 female patients in the study. Each patient in the study was asymptomatic and the lesions were discovered incidentally by CT scan, which was done for other reasons. Each patient was then evaluated by CT angiography and endoscopic studies, which included ERCP, EUS, biopsy, and cytology. (Table 1)

**Table 1:** Patient summary

Patient	Gender	Age	Pathology	**PMHx**	**Complications**
1	M	77	IPMT	CAD, COPD	Pneumonia

2	F	68	IPMT	DM, HTN, obesity	Local wound infection

3	F	71	Mucinous cystic neoplasm	HTN	None

4	M	57	Lymphoid cystic neoplasm	COPD, obesity	Local wound infection

5	F	34	Neuroendocrine tumor	None	None

6	M	66	Neuroendocrine tumor	None	Local wound seroma

7	F	46	Clear cell adenoma	None	None

8	F	49	Neuroendocrine tumor	None	None

9	M	43	Neuroendocrine Tumor	Obesity	None

10	M	59	Neuroendocrine tumor	HTN	None

### Technical aspects

**Figure 1:**
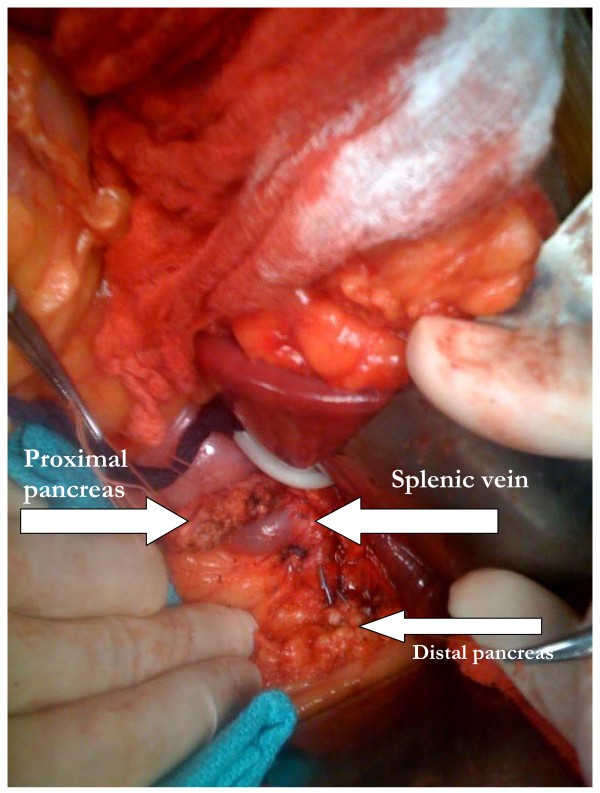
**Operative site after removal of central portion of the pancreas**.

**Figure 2:**
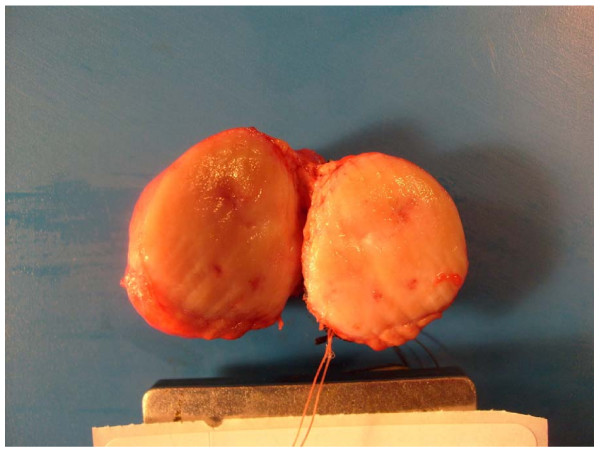
**Gross section of the tumor, diameter 2.8 cm**.

Each operation was performed through an upper midline incision. The stomach is retracted downwards while the gastro-hepatic omentum is incised exposing the neck, body, and a portion of the tail of the pancreas. The gastro-colic omentum is dissected as needed. If necessary, the stomach can be retracted superiorly while the transverse colon is retracted downwards and this facilitates exposure of the lower border of the pancreas and dissection of the superior mesenteric vein (SMV) behind the pancreas. Stay sutures are placed on either side of the lesion in the superior and inferior aspect of the pancreas. This facilitates dissection from the SMV and the stay sutures also help to control the transverse pancreatic vessels as well. Once the SMV is completely dissected from the pancreas, the distal margin of pancreas is transected, while protecting the SMV. The specimen is then excised by transecting the proximal margin. (Figure 1) The lesion is then sent to pathology to be evaluated for margins by frozen section, an example is seen in figure 2. The transected pancreas is oversown after ligating both ends of the transected pancreatic duct. The pancreatic duct is suture ligated with a 4-0 vicryl suture and then the transected pancreas is oversewn with a running 4-0 prolene suture, imbricating the pancreatic capsule. A drain is placed and the abdomen is closed in standard fashion. The drains were removed upon discharge because there were no fistulas in our group.

## Results

The resected lesions included a branch IPMN in 2 patients, a mucinous cyst, a lymphoid cyst, five neuroendocrine tumors, and a clear cell adenoma (Table 1). The mean operative time was 73.5 ± 10 minutes, and the estimated blood loss was 164 ± 89 ml. There were no mortalities in the study. The postoperative length of stay (LOS) was 5.9 ± 9.5 days (range 4 to 30); however, this was skewed by one patient with COPD, who had pneumonia postoperatively and was hospitalized for 30 days. The LOS for the other patients in the study was 4.8 ± 0.75 days. Other postoperative complications included a superficial wound infection in 2 patients, and a wound seroma in one patient. These three patients were also obese.

## Discussion

Central pancreatectomy has a unique application in certain patients with focal, chronic pancreatitis and trauma. It is utilized mostly for benign and low-grade malignant lesions in the neck of the pancreas [5,9-12]. The potential benefit of CP is to preserve pancreatic function and the spleen by limiting resection of normal parenchyma [2]. Diabetes mellitus (DM) occurs in 20% of patients following distal pancreatectomy [13-15]. Endocrine insufficiency is more frequent in patients with chronic pancreatitis and approaches 50% within 5 years after distal pancreatectomy (DP). Endocrine and exocrine insufficiency depends on residual function of the pancreas and the severity of pancreatitis [16]. The long-term risk of DM after pancreatic resection is greater after distal resection of the pancreas rather than after CP (11%, vs 50%) [8], particularly in an already diseased gland. The benefits of CP are obvious regarding pancreatic and splenic function [3,6,17]. Preservation of splenic function in the pediatric population may be important. Most CPs have utilized a pancreatico-jejunal or pancreatico-gastric anastomosis to the distal pancreas. The incidence of postoperative fistula in patients with a CP anastomosis ranges from 8% to 40% with a re-operative rate as high as 12% [2,9,18-20]. The incidence of a pancreatic leak after CP and pancreatic anastomosis is summarized in Table 2.

**Table 2:** Postoperative Results; Literature Review

	**N**	**Type of pancreatic** **anastomosis**	**Fistula rate (number and percentage)**	**Other Complications**
**Allendorf **[8]	26	Pancreatico- gastrostomy	2/26 (7.7%)	None

**Efron **[9]	14	pancreaticogastrostomy	5/14 (36%)	

**Roggin **[6]	10	Central Pancreatectomy	3/10 (%30)	1

**Christein**, [2]	8	Roux-en-Y pancreaticojejunostomy	5/8 (63%)	25 re-operation for bleeding

**Shimada **[3]	10	Roux-en-Y pancreaticojejunostomy	5/10 (50%)	

**Ocuin **[4]	31	Central	38%	Exocrine/
		pancreatectomy		Endocrine
		(CP) n = 13		10% 57%
		extended left pancreatectomy	17%	
		(ELP) n = 18		27% 10%

**Goldstein **[10]	12	Roux-en-Y pancreaticogastrostomy	0/12, 0%	2/12 had endocrine insufficiency

**Warshaw **[12]	12	Roux-en-Y pancreaticojejunostomy	2/12,	One patent with gastric emptying

**Sauvanet **[20]	53	Roux-en-Y pancreaticojejunostomy	16/53, (30%)	40

**Adham **[19]	50	Roux-en-Y pancreaticojejunostomy	11/38, 22%	8% fistula (14% intra-abdominal collection) 6% bleeding

**Fahy **[24]	51	Distal pancreatectomy	11/51, (26%)	

**Johnson **[25]	8	Roux-en-Y pancreaticojejunostomy	0	No post-op endocrine insufficiency

We suspect the relative frequency of a pancreatic fistula after CP is due to a small pancreatic duct and a normal soft distal gland. These two factors (a small duct and soft parenchyma) account for a higher fistula rate after pancreatico-duodenal resection (PDR). This is our reasoning for omitting a pancreatico-enteric anastomosis during CP. In our experience, the distal pancreatic tissue is usually normal and the duct is small in diameter. The indications for CP in chronic pancreatitis are few since focal pancreatitis confined to the neck of gland is unusual. CP may be technically more difficult because of chronic inflammation in these patients [2]. Furthermore, in patients with a pancreaticogastrostomy, fistula rates aside, exocrine function may not be preserved. Pancreatic enzymes, particularly lipase, are inactivated in an acidic environment [21-23]. Our series of 10 patients supports the value of resection without anastomosis in a short follow up period. To date, none of the patients in the study have developed any endocrine or exocrine deficiencies. So far, the morbidity of a pancreatic leak is removed while exocrine function is preserved in the head and neck and endocrine function remains in both segments of pancreas when using central pancreatectomy without an anastomosis.

## Conclusion

CP without an anastomosis may reduce the morbidity and length of hospital stay compared to patients undergoing CP with an anastomosis. It has been shown to be a safe, effective procedure which does not compromise pancreatic function.

## Competing interests

The authors declare that they have no competing interests.

## Authors' contributions

MW was the lead author and surgeon for all of the patients. SNM gathered information and contributed to writing of the paper. FK and WB were the GI doctors who contributed patients and information on the patients. AVC reviewed paper and technique of surgery. AC was the co-surgeon on the cases. AP contributed to the literature review.
